# Radiosensitization effect of iridium (III) complex on lung cancer cells via mitochondria apoptosis pathway

**DOI:** 10.3389/fphar.2025.1562228

**Published:** 2025-03-27

**Authors:** Yuru Pang, Qiqi Meng, Yangchen Cui, Shiyi Liu, Huihui Jiang, Chenlan Xu, Yan An, Yang Jiao, Qi Zhang, Jihua Nie

**Affiliations:** ^1^ Department of Toxicology, School of Public Health, Medical College of Soochow University, Suzhou, Jiangsu, China; ^2^ State Key Laboratory of Radiation Medicine and Protection, School of Radiation Medicine and Protection, Collaborative Innovation Center of Radiological Medicine of Jiangsu Higher Education Institutions, Soochow University, Suzhou, China; ^3^ Key Laboratory of Radiation Damage and Treatment of Jiangsu Provincial Universities and Colleges, Collaborative Innovation Center of Radiological Medicine of Jiangsu Higher Education Institutions, Soochow University, Suzhou, China; ^4^ Key Laboratory of Radiation Medicine and Protection, Soochow University, Suzhou, China

**Keywords:** iridium (III) complexes, lung cancer, mitochondria apoptosis pathway, radiation sensitization, ROS

## Abstract

**Background:**

Lung cancer is the leading cause of cancer-related death in the worldwide. Although cisplatin and other platinum-based drugs are widely used as radiosensitizers in radiotherapy and considered the first-line treatment for advanced lung cancer, their clinical utility is often limited by drug resistance and severe cytotoxic side effects. In recent years, iridium-based complexes and other transition metal cation complexes with similar structural properties have garnered increasing research interest due to their potential anticancer properties.

**Methods:**

Recently, we synthesized a novel iridium (III) complex (Ir-1) and evaluated its safety and stability. The present study aimed to identify Ir-1 with potent anticancer activity by assessing its cytotoxic effects on lung cancer cells in vitro. Additionally, it investigated Ir-1's radiosensitizing efficacy and the underlying mechanisms.

**Results:**

The results demonstrated that Ir-1 exhibited significant radiosensitizing effects on lung cancer cells. Ir-1 effectively reduced cell viability and colony formation, arrested the cell cycle at the G2/M phase, inhibited cell migration and invasion, decreased mitochondrial membrane potential, and increased reactive oxygen species (ROS) generation in lung cancer cells. Importantly, these cytotoxic effects were selective, with minimal impact on normal cells. Mechanistic studies showed that Ir-1 enhanced radiation-induced cancer cell death by disrupting mitochondrial function and activating the mitochondrial apoptotic pathway. This was evidenced by upregulated expression levels of Bax, Cytochrome c (Cyt-C), and Caspase9 proteins, along with reduced level of Bcl-2 protein. Notably, the addition of a Cyt-C inhibitor significantly reduced the expression of Cyt-C and Caspase9 proteins. Similarly, treatment with the Caspase9 inhibitor Z-LEHD-FMK also reduced Caspase9 protein level.

**Conclusion:**

This study provides robust evidence that Ir-1 is a promising and safe radiosensitizer for lung cancer therapy. Its ability to enhance radiation-induced cytotoxicity through mitochondrial dysfunction and activation of apoptotic pathways highlights its potential for clinical application.

## 1 Introduction

Lung cancer is a malignant tumor with increasing morbidity and mortality worldwide (Xia et al., 2022). Clinically, the treatment strategies for lung cancer are determined by factors such as disease stage, histological type, and the patient’s overall health status. Available options include surgical resection, radiation therapy, chemotherapy, targeted therapy, and various combination therapies. Among these, targeted therapy has introduced an innovative approach to cancer treatment, offering the advantage of minimizing side effects commonly associated with cytotoxic chemotherapy drugs (van den Bulk et al., 2018). However, it still has certain limitations (Zafar et al., 2021). Currently, for most patients with lung cancer, radiotherapy combined with sensitizers remains a cornerstone treatment option. Especially, chemoradiotherapy with platinum drugs is clinically recommended for the stage III-IIIA disease (Alexander et al., 2020). Since Rosenberg discovered cisplatin in the 1960s, platinum-based chemotherapy has played a pivotal role in cancer treatment. However, its clinical effectiveness is often limited by the development of drug resistance (Okamoto et al., 2020) and dose-limiting toxicities, including neurotoxicity, nephrotoxicity, and hepatotoxicity (Rosenberg et al., 1969; Santos et al., 2020). Researchers are committed to finding new metal based anticancer agents that are expected to replace platinum drugs with improved selectivity and safety. Transition metal cation complexes have emerged as promising candidates in this context (Ni et al., 2022). Previous studies have demonstrated that certain metal complexes (such as iridium, ruthenium, copper, and nickel) exhibit anticancer potential by interacting with key organelles such as the nucleus, mitochondria, and endoplasmic reticulum (Luo et al., 2021; Giorgi et al., 2022).

Iridium belongs to the same family as platinum and is located in the third row of transition metals, directly adjacent to platinum, resulting in many similar properties to those of platinum (Marloye et al., 2016). Compared with platinum, which typically adopts a planar structure, iridium shows the advantage of structural diversity, enabling various ligand modifications through multiple coordination geometries (Konkankit et al., 2018). Liu et al. reported that Ir-Cpx, an organometallic iridium (III) complex with a semi sandwich structure, has cytotoxicity against A2780 cells (Liu et al., 2011). Similarly, Conesa et al. demonstrated that ACC25, a new iridium (III) complex with a semi sandwich structure protected by two chelating rings, exhibited anti-proliferative effects on MCF7 breast cancer cells, including mitochondria damage and compromising other organelles (Conesa et al., 2020). Several studies have highlighted that iridium (III) complexes could exhibit different properties by adjusting the ligands of iridium. Bis(2-methyldibenzo [f,h]quinoxaline) (acetylacetonate) contains a lipophilic aromatic group that promotes cellular uptake of its iridium (III) complex when dissolved in DMSO (Cao et al., 2013; Liao et al., 2018). We previously synthesized the iridium (III) complex Ir (MDQ)_2_ (acac) and demonstrated its potential as a novel probe for tracking mouse neural stem cells (Xu et al., 2020), with excellent biocompatibility and photostability (Li et al., 2019).

In this study, we confirmed the inhibitory effects of iridium (III) complex on lung cancer cells, particularly its ability to enhance the radiation-induced inhibition of lung cancer cells. Furthermore, we investigated the potential mechanism by which iridium complexes promote mitochondrial apoptosis through the activation of ROS signaling pathways.

## 2 Materials and methods

### 2.1 Materials and chemicals

**FIGURE 1:**
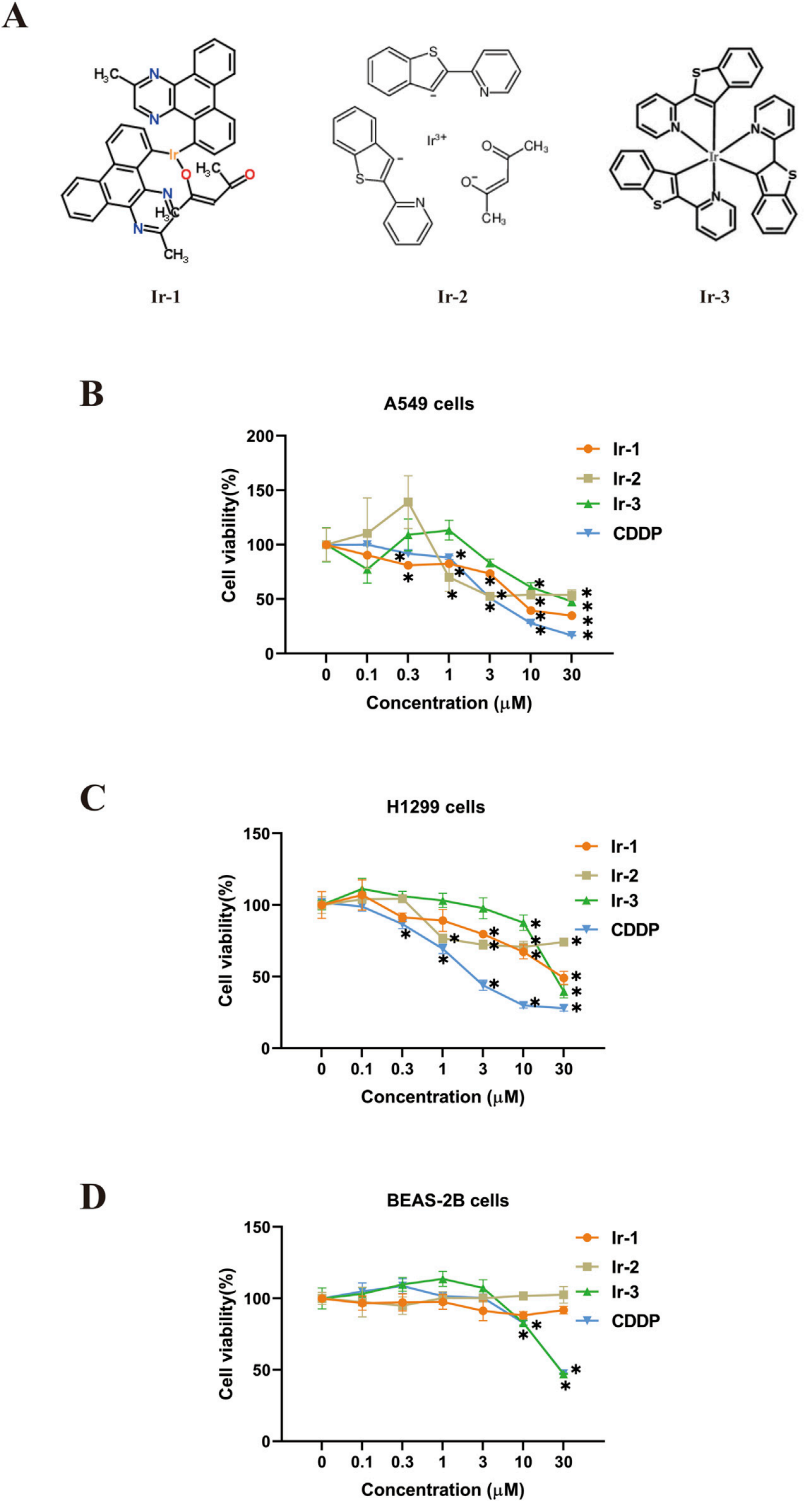
Effects of iridium (III) complexes on cell viability. **(A)**, Structures of three Iridium (III) complexes. **(B, C)**, treatment with Ir-1, Ir-2, Ir-3 and positive control CDDP of at (0.1, 0.3, 1, 3, 10, and 30 μM) to lung cancer cells (B, A549 cell; C, H1299) and normal BEAS-2B cells **(D)** for 24 h. Data shown as mean ± SD, n = 3. *p < 0.05 compared with 0 μM.

Ir-1 complex (Ir-1) [Ir (MDQ)2 (acac)] was synthesized and characterized by Dan Li from our research group (Li et al., 2019). Ir-2 [Ir (btpy)2 (acac)] and Ir-3 [Ir (btpy)3] were purchased from Jilin OLED Material Tech Co., Ltd. (Jilin, China, CAS numbers 343978-79-0 and 405289-74-9). Their chemical formulas were shown in Figure 1A. CDDP was used as a positive control drug purchased from Shanghai Aladdin Biochemical Technology Co., Ltd. (Shanghai, China, CAS number 15663-27-1). All the chemicals and reagents were of analytical grade. Ir-1, known as Bis (2-methyldifenzo [f, h] quinoxaline) (acetylacetonate) Iridium (III), was reported to be mostly used in two-photon imaging or OLED diodes and had anticancer activity (Li et al., 2019; Xu et al., 2020). Ir-2, known as Bis [2-(2′-benzothienyl) pyridinato-N, C3'] (acetylacetonato) Iridium (III), was reported to have certain anticancer activity (Zhang et al., 2020; Zhang et al., 2021; Yang et al., 2022). Ir-3, known as Tris [2-(benzo[b]thiophen-2-yl) pyridine-C3, N] Iridium (III), could be used as an oxygen probe (Fischer et al., 2009). The antibodies for Bcl-2, Bax, Cyt-C, Caspase9 and Caspase3 were purchased from Abcam (Cambridge, MA, United States). The antibodies for β-tubulin and GAPDH were purchased from Abbkine Biotechnology Co., Ltd. (Wuhan, China). Cyt-C inhibitor was purchased from APExBIO (Houston, TX, United States). Z-LEHD-FMK was purchased from MedChem Express (Monmouth Junction, NJ, United States).

### 2.2 Cell culture

Human non-small cell lung cancer (NSCLC) cells (H1299) and human normal lung epithelial cells (BEAS-2B) were obtained from the Shanghai National Collection of Authenticated Cell Cultures (Shanghai, China). Human NSCLC cells (A549) were obtained from Wuhan Procell Life Science & Technology Co., Ltd. (Wuhan, China). All the cells were cultured in Dulbecco’s Modified Eagle Medium (DMEM) supplemented with 10% fetal bovine serum (FBS) and 1% penicillin/streptomycin. The cells were maintained under standard cell culture conditions at 37°C and 5% CO_2_ in a humid environment. Attached cells were harvested using trypsin and re-suspended in a serum-containing medium before used in cell cryopreservation, passage and the assays, as described below.

### 2.3 Cell cytotoxicity

The cell cytotoxicity was measured by CCK-8 assay. Cells were seeded into 96-well plates at a density of 1 × 10^4^ cells per well and cultured in 200 μL of DMEM complete medium. Then, different concentrations (0 μM, 0.1 μM, 0.3 μM, 1 μM, 3 μM, 10 μM, and 30 μM) of Ir-1, Ir-2, Ir-3, and CDDP dissolved in DMSO were treated on attached cells for 24 h, followed by addition of the mixture of CCK-8 and culture medium in a 1:9 ratio to each well and incubation at 37°C for 1–2 h. The absorbance (OD value) of the cells at 450 nm was measured using the ELISA reader. The data were analyzed to calculate cell viability at different drug concentrations.

### 2.4 Clonogenic assay

A549, H1299 and BEAS-2B cells were seeded in 6-well plates at a respective density of 200, 400, and 800 cells treated with 6 μM of Ir-1 for 24 h. The cells were exposed to 0, 2, 4, and 6Gy irradiation, and then were cultured in a 5% CO_2_ incubator at 37°C for 14 days. After fixed with 4% paraformaldehyde and stained with crystal violet, colonies containing ≥50 cells were counted under a microscope. The experiment was repeated three times to calculate the clone survival fraction and radiation sensitization ratio.

### 2.5 Detection of apoptosis

Flow cytometry analysis was performed to assess the percentage of apoptotic cells following treatment with Ir-1 and radiation, using the Annexin V-PE/7-AAD apoptosis detection kit. Cells were cultured into 6-well plates at a density of 1 × 10^5^ cells per well in cell incubator for 24 h. After the pre-treatment (6 μM Ir-1), cells were exposed to 0, 2, 4, and 6 Gy irradiation to screen for optimal radiation dose for cell apoptosis. Subsequently, the cells were harvested, washed twice with phosphate-buffered saline (PBS), and re-suspended in 100 μL of buffer, which was then added with 5 μL of Annexin V-PE and 5 μL of 7-AAD, and then incubated at room temperature in the dark for 15–20 min. Flow cytometry was then conducted to evaluate the apoptosis rates under different treatment conditions.

### 2.6 Cell cycle analysis

To determine the effect of Ir-1 and radiation treatment on the cell cycle regulation, cell cycle analysis was performed as follows: cells were cultured into 6-well plates at a density of 1 × 10^6^ cells per well and incubated overnight. After 24 h, the cells were divided into Control group, Ir-1 group, 6 Gy group, and Ir-1+6 Gy group. Each group consisted of three replicate wells. Cells were pre-treated with the respective drugs for 24 h, followed by 6 Gy irradiation. Samples were collected and then centrifuged at 1,500 rpm for 3 min and fixed with 70% ethanol overnight in 4°C. The cells were washed with cold PBS to remove ethanol content and incubated at 37°C with RNase A. The cells were then stained with the propidium iodide (PI) for 30 min. Flow cytometry analysis was performed within 24 h to assess cell cycle distribution.

### 2.7 Wound healing assay

To assess the impact of Ir-1 and radiation on lung cancer cells migration, a wound-healing assay was performed using A549 and H1299 cells. Cells were divided into Control group, Ir-1 group, 6 Gy group, and Ir-1+6 Gy group. The cells were seeded in 6-well plates and allowed to reach a confluent state, then a single scratch was made using a sterile 200 μL pipette tip. The cells were then washed to remove debris and incubated with FBS-free culture medium. After scratch cleaning, images of the scratches were captured at 0 and 24 h with an inverted fluorescence microscope. The results were statistically analyzed by ImageJ. The effects of Ir-1 and 6 Gy irradiation on the migration ability of lung cancer cells were evaluated.

### 2.8 Transwell assay

To assess the effect of Ir-1 and radiation on lung cancer cells invasion ability, a Transwell assay was performed using 24-well Matrigel invasion chambers. Each Transwell insert was fitted with a polycarbonate membrane featuring 8 μm pores, precoated with 100 μL of Matrigel, and incubated at 37°C for 30 min to 1 h to facilitate gel solidification. A549 cells and H1299 cells were divided into Control group, Ir-1 group, 6 Gy group, and Ir-1+6 Gy group. The cells were pre-treated with Ir-1 for 24 h and 6 Gy irradiation. After 24 h post-irradiation, cells were harvested and reseeded into the upper chamber at a density of 1 × 10^5^ cells/mL in 200 μL of serum-free RPMI-1640 medium. The lower chamber contained 800 μL of RPMI-1640 supplemented with 20% FBS. After incubation for 48 h, the medium was discarded. The cells that had invaded the lower surface of the membrane were fixed in 4% paraformaldehyde for 20–30 min and stained with 0.1% crystal violet for 10–20 min. The cell membrane penetration was observed under a fluorescence microscope and the number of invaded cells was quantified for statistical analysis.

### 2.9 Mitochondrial membrane potential assay

To examine the effect of Ir-1 and radiation on lung cancer cells mitochondrial membrane potential (ΔΨ_m_), the mitochondrial membrane potential assay was carried out using the Mitochondrial Membrane Assay Kit and JC-1 (5,5′,6,6′-Tetrachloro-1,1′,3,3′-tetraethyl-imidacarbocyanine iodide) as fluorescence probe. A549, H1299, and BEAS-2B cells were seeded into a 6-well plate at a density of 1 ×10^5^ cells per well and subjected to the same pretreatment conditions as described above. After 24 h incubation, cells were harvested and stained with JC-1 dye (green, λ_ex_ = 490 nm, λ_em_ = 530 nm, red, λ_ex_ = 525 nm, λ_em_ = 590 nm) in dark for 20 min at 37°C. For the positive control, the cells were exposed to carbonyl cyanide m-chlorophenylhydrazone (CCCP) for 20 min.

### 2.10 Intracellular ROS assay

The intracellular reactive oxygen species (ROS) generation induced by Ir-1 and radiation was analyzed using the Reactive Oxygen Species Assay Kit and flow cytometry. A549, H1299 and BEAS-2B cells were seeded into a 6-well plate at a density of 1 × 10^5^ cells per well, following the same pretreatment as described above. The cells were then collected and re-suspended in a working solution containing 10 μM of DCFH-DA (λ_ex_ = 488 nm, λ_em_ = 525 nm) for 20 min at 37°C in the dark to avoid light-induced ROS production. The samples were taken out every 4–5 min and mixed to ensure full contact between the cells and the probe. For the positive control, the cells were exposed to ROSUP for 20 min. The samples were then analyzed by flow cytometry and data analysis was conducted using GraphPad Prism 8.0 software.

### 2.11 Western blot analysis

Cells were harvested as scheduled after treatment under different conditions with Cyt-C inhibitor or Z-LEHD-FMK and rinsed three times with PBS. The cells were then lysed on ice using a lysis buffer containing a protease inhibitor cocktail and radioimmunoprecipitation assay (RIPA) buffer. After 20 min, the cells were scraped and the lysate collected in an Eppendorf tube was cleared by centrifugation at 12,000 rpm for 15 min at 4°C to remove debris. The protein concentration in the supernatant was determined by the Bicinchoninic Acid Assay. For Western blotting, equal amounts of total proteins were separated on 12.5% sodium dodecyl sulfate polyacrylamide gel electrophoresis (SDS-PAGE) and transferred onto a polyvinylidene fluoride (PVDF) membrane for 2 h. The membranes were blocked with 5% skimmed milk at room temperature for 1 h, followed by washing with PBST for 10 min. The membranes were incubated with primary antibodies at 4°C for overnight. On the next day, after washed for 3 times, the membranes were incubated with HRP-conjugated secondary antibody for 1 h at room temperature. Immunoreactive bands were visualized using an enhanced chemiluminescence system.

### 2.12 Statistical analysis

Graphpad Prism 8.0 and ImageJ were used in statistical analysis. Numerical data were presented as mean ± standard deviation (SD) from at least three experiments. The pairwise comparison of different group results were made using SPSS 19.0 for SNK-q test. The value of *P < 0.05* was considered statistically significant.

## 3 Results

### 3.1 Iridium (III) complexes reduced lung cancer cells viability

The effects of the Iridium (III) complexes on the viability of lung cancer cells were evaluated using the CCK-8 assay and the IC50 values were determined from the assay results. Results showed that Ir-1, Ir-2 and Ir-3 reduced cancer cell viability in a dose-dependent manner (Figures 1B–D; Table 1). Specifically, Ir-1 reduced viability of A549 cells and H1299 cells, while showing no effect on normal epithelial MEAS-2B cells. Ir-2 selectively reduced the viability of A549 cells without affecting H1299 cells. In contrast, Ir-3 reduced the viability of A549, H1299, and MEAS-2B cells. CDDP was used as a positive control in all experiments. Based on these results, Ir-1 was chosen for the subsequent experiments. To investigate its potential to enhance the sensitivity of cancer cells to radiotherapy, we selected the concentration of Ir-1 that achieved cells viability at 75% (6 μM for A549 and 25 μM for H1299) for subsequent experiments. A concentration of 6.5 μM CDDP was used as a positive control in these experiments.

**TABLE 1:** IC_50_ (μM) values of the iridium (III) complexes and CDDP.

IC_50_ (μM)	A549	H1299	BEAS-2B
Ir-1	11.16 ± 0.51	53.89 ± 5.63	>200
Ir-2	14.46 ± 2.84	>200	>200
Ir-3	15.31 ± 0.57	25.61 ± 0.23	23.93 ± 0.92
CDDP	12.94 ± 0.22	6.52 ± 0.39	28.13 ± 0.58

### 3.2 Ir-1 increased radiation-induced inhibition of colony formation in lung cancer cells

**FIGURE 2:**
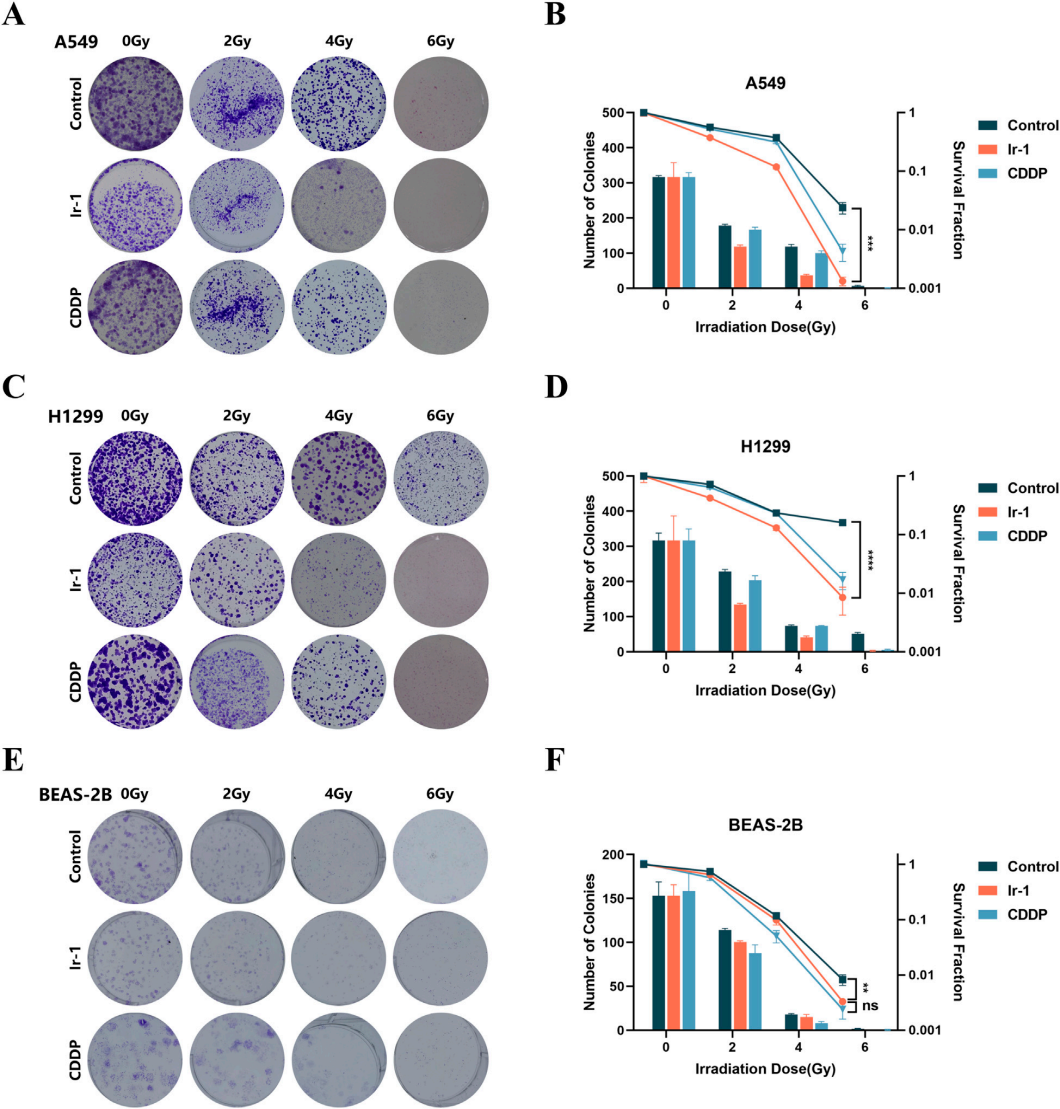
Ir-1 increased radiation-induced inhibition of colony formation in lung cancer cells. The A549 **(A, B)**, H1299 **(C, D)** and BEAS-2B **(E, F)** cells were treated with Ir-1 and CDDP for 24 h and exposure to 6 Gy radiation. Survival fraction was used to evaluate cell radiosensitivity. Data shown as mean ± SD, n = 3. **p < 0.01, ***p < 0.001, ****p < 0.0001.

Next, we examined the synergistic effects of Ir-1 and radiation therapy in lung cancer cells. Firstly, the inhibitory effect of Ir-1 in combination with different radiation doses (0, 2, 4, 6 Gy) on colony formation was evaluated in A549, H1299, and BEAS-2B cells. Figures 2A, B) illustrates that Ir-1 potentiated the inhibitory effect of radiation on colony formation in A549 cells. The results indicate that Ir-1 significantly enhances the inhibitory effect at a radiation dose of 6 Gy. A similar enhancement was observed in H1299 cells, as shown in Figures 2C, D, where Ir-1 significantly increased the radiation-induced inhibition of colony formation at 6 Gy. The sensitive enhancement ratio (SER) of Ir-1 in A549 cells (1.592) and H1299 cells (1.191) were higher than compared to CDDP in the respective cell lines (1.099 and 1.138). Additionally, Ir-1 also enhanced the radiation-induced inhibition of colony formation in BEAS-2B cells (Figures 2E, F), the SER of Ir-1 in BEAS-2B cells was 1.003 which was lower than that in A549 and H1299 cells. Based on these results, 6 Gy was selected as the optimal radiation dose for subsequent experiments.

### 3.3 Ir-1 increased radiation-induced apoptosis in lung cancer cells

**FIGURE 3:**
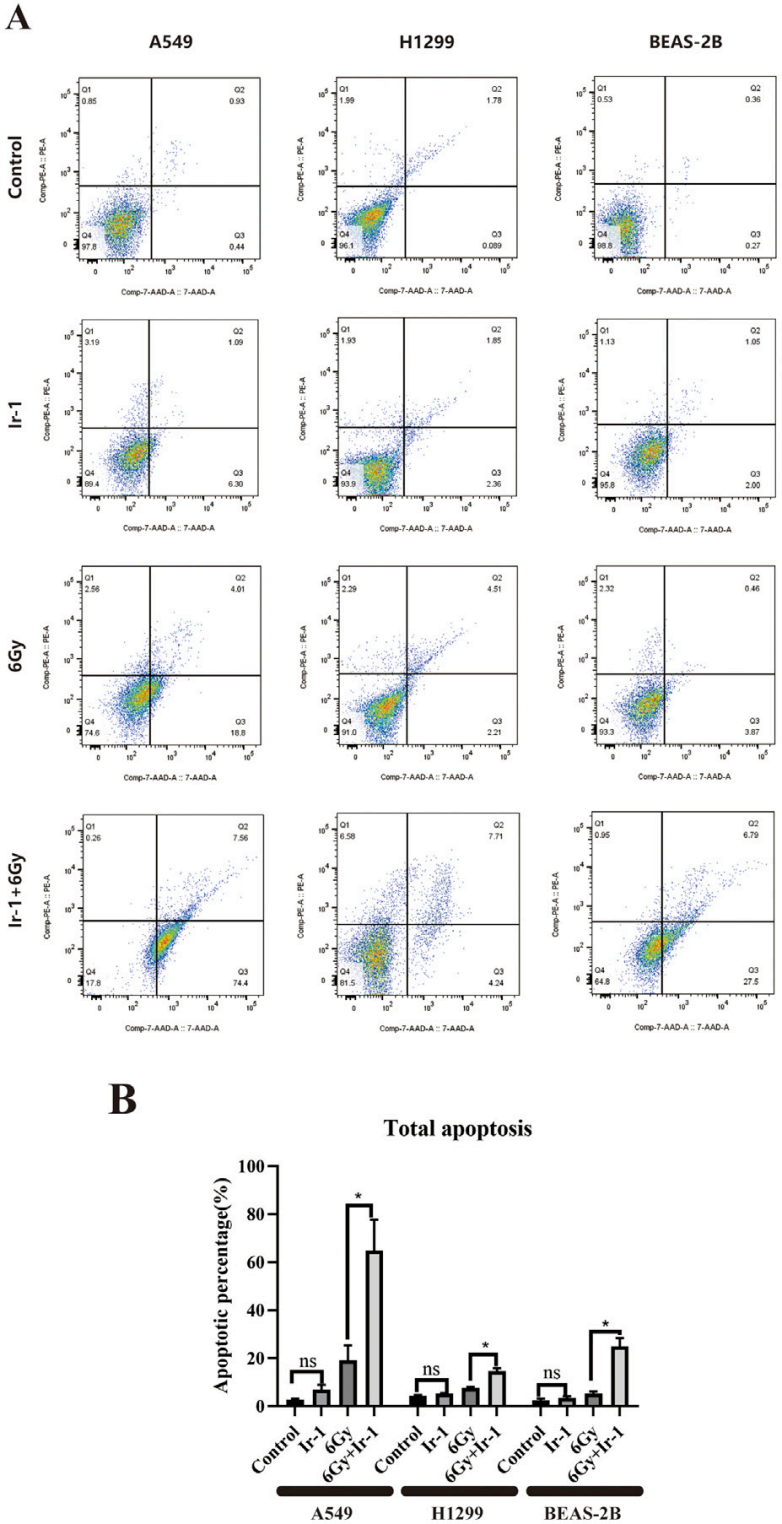
Ir-1 increased radiation-induced apoptosis in lung cancer cells. **(A)**, Representative flow cytometry results for A549, H1299 and BEAS-2B cells. **(B)**, The data analysis from flow cytometry results. Data shown as mean ± SD, n = 3. *P < 0.05.

Following, we examined whether Ir-1 could enhance radiation-induced lung cancer cells apoptosis. The results indicated that in both A549 cells and H1299 cells, the numbers of apoptotic cells in the Ir-1 + 6 Gy radiation group were significantly higher compared to the 6 Gy radiation group (Figures 3A, B).

### 3.4 Ir-1 increased radiation-induced cell cycle arrested in lung cancer cells

**FIGURE 4:**
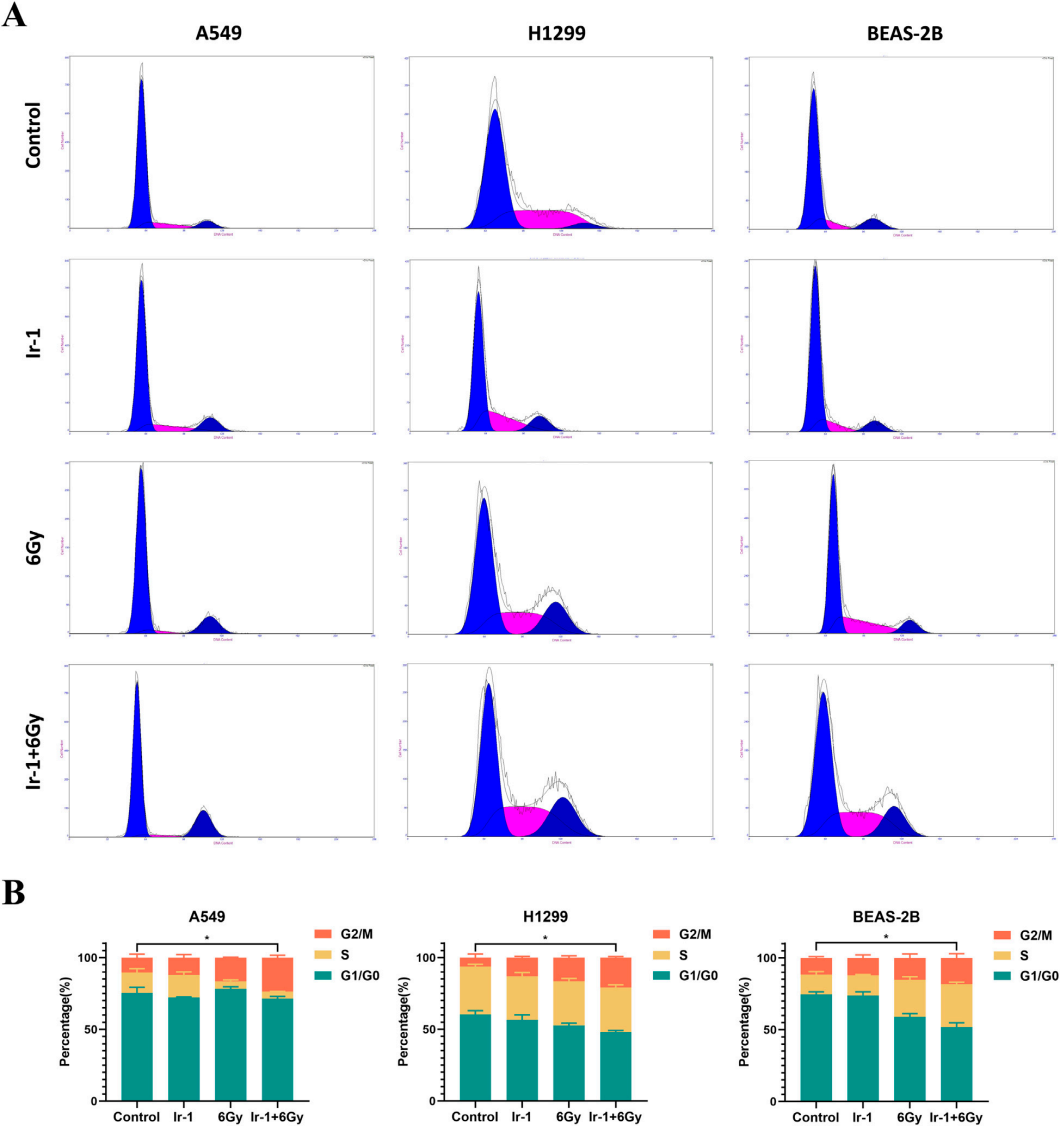
Ir-1 increased radiation-induced cell cycle arrested in lung cancer cells. **(A)**, Representative flow cytometry results of cell cycle distribution in A549, H1299, and BEAS-2B cells following 24-hour treatment with Ir-1 or CDDP, with or without 6 Gy irradiation. **(B)**, Cell cycle distribution analysis based on **(A)**. Data shown as mean ± SD, n = 3.

We then examined whether Ir-1 combined with radiation influenced the cell cycle distribution of lung cancer cells. As shown in Figure 4, the cell cycle distribution of A549 cells and H1299 cells was significantly altered in the Ir-1 + 6 Gy group, reflecting that an increase at G2/M phase in Ir-1+6 Gy group compared with other groups. In the 6 Gy group, the proportions of A549 cells in the G0/G1, S, and G2/M phase were respectively 78.24% ± 1.18%, 5.17% ± 0.98%, and 16.59% ± 0.28%, respectively. In the Ir-1 + 6 Gy group, these proportions changed to 71.48% ± 1.25%, 4.37% ± 0.24%, and 23.79% ± 1.36%, indicating a decrease of approximately 6.76% in the G0/G1 phase and an increase of approximately 7.2% in the G2/M phase. The similar results were found in H1299 cells. In BEAS-2B cells, only slight changes in G0/G1, S and G2/M phase were observed between 6Gy group and Ir-1 + 6 Gy group.

### 3.5 Ir-1 enhanced radiation-induced inhibitory effect of migration and invasion in lung cancer cells

**FIGURE 5:**
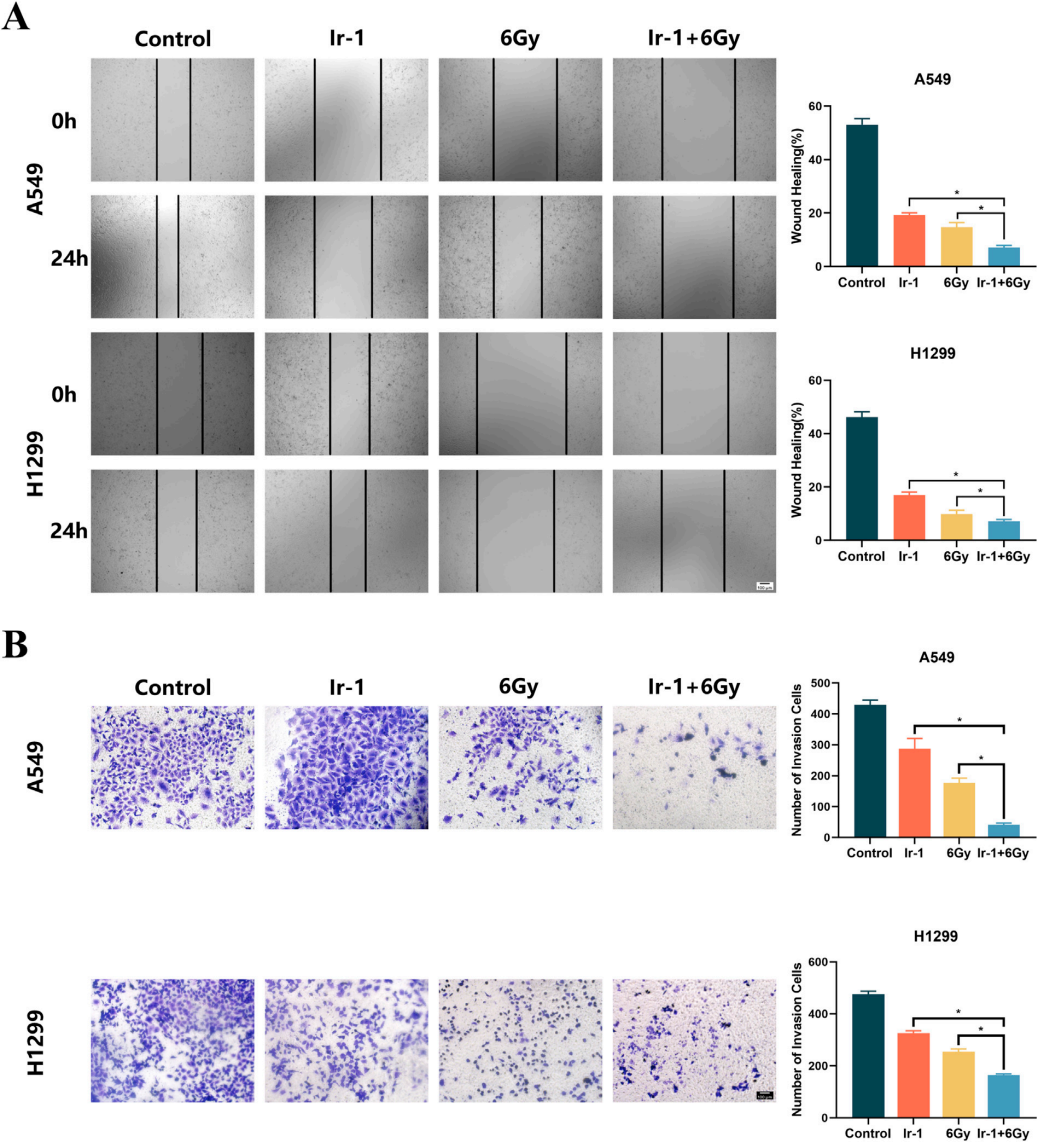
Ir-1 enhanced radiation-induced inhibition of migration and invasion in lung cancer cells. **(A)**, Ir-1 increased radiation-induced inhibition of migration in A549 and H1299 cells. **(B)**, Ir-1 increased radiation-induced inhibition of invasion in A549 and H1299 cells. Data shown as mean ± SD, n = 3. *P < 0.05.

The wound healing assay and Transwell assay were conducted to examine whether Ir-1 enhances the inhibitory effects of radiation on cancer cell migration and invasion. Figure 5A shows the wound healing assay results. The migration rate of A549 cells and H1299 cells in the Ir-1+6Gy group was significantly lower than that in the 6Gy group. Figure 5B shows Transwell assay results. The number of A549 cells and H1299 cells that penetrated the membrane in the Ir-1+6Gy group was significantly lower than that in the 6Gy group. These results indicate that Ir-1 complex enhances the radiation-induced inhibitory effect on lung cancer cell migration and invasion.

### 3.6 Ir-1 enhanced radiation-induced reduction of the mitochondrial membrane potential in lung cancer cells

**FIGURE 6:**
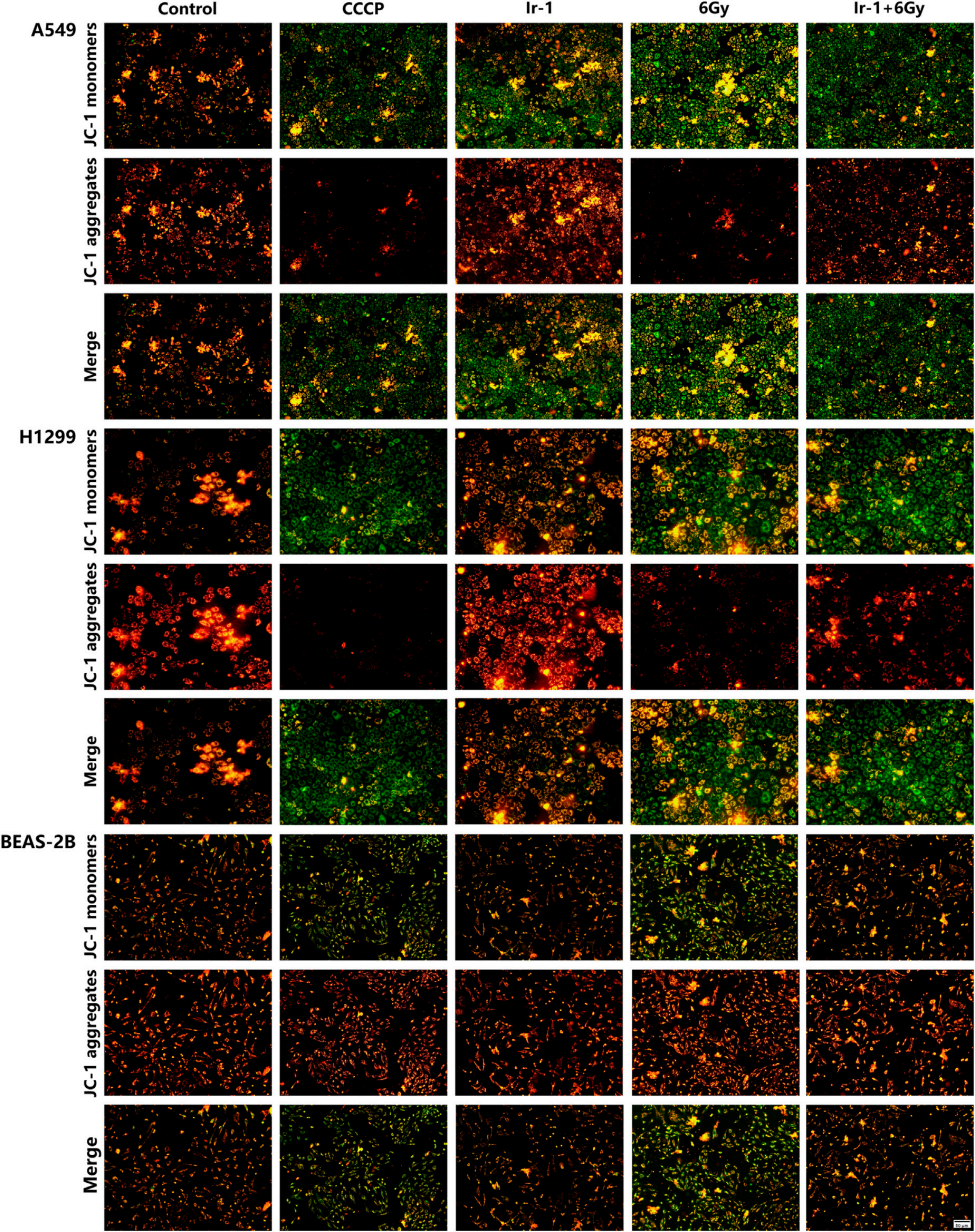
Ir-1 enhanced radiation-induced reduction the mitochondrial membrane potential of lung cancer cells.

To explore the underlying mechanism of Ir-1 action, we analyzed the changes in mitochondrial membrane potential levels using JC-1 dye as an indicator. As shown in Figure 6, in the Ir-1+6 Gy group of both A549 cells and H1299 cells, JC-1 dye exhibited brighter green fluorescence (monomers corresponding to low mitochondrial membrane potential) and weaker red fluorescence (aggregates at the high mitochondrial membrane potential) compared with other groups, especially the 6 Gy group. This suggests a significant reduction in mitochondrial membrane potential when Ir-1 is combined with radiation. In contrast, BEAS-2B cells showed no significant green fluorescence in the Ir-1 or Ir-1+6 Gy group, indicating that Ir-1 and radiation did not notably affect the mitochondrial membrane potential in normal lung epithelial cells. These findings suggest that the combination of Ir-1 and 6 Gy irradiation specifically reduces the mitochondrial membrane potential in lung cancer cells, which may trigger early apoptosis.

### 3.7 Ir-1 enhanced radiation-induced the ROS generation in lung cancer cells

**FIGURE 7:**
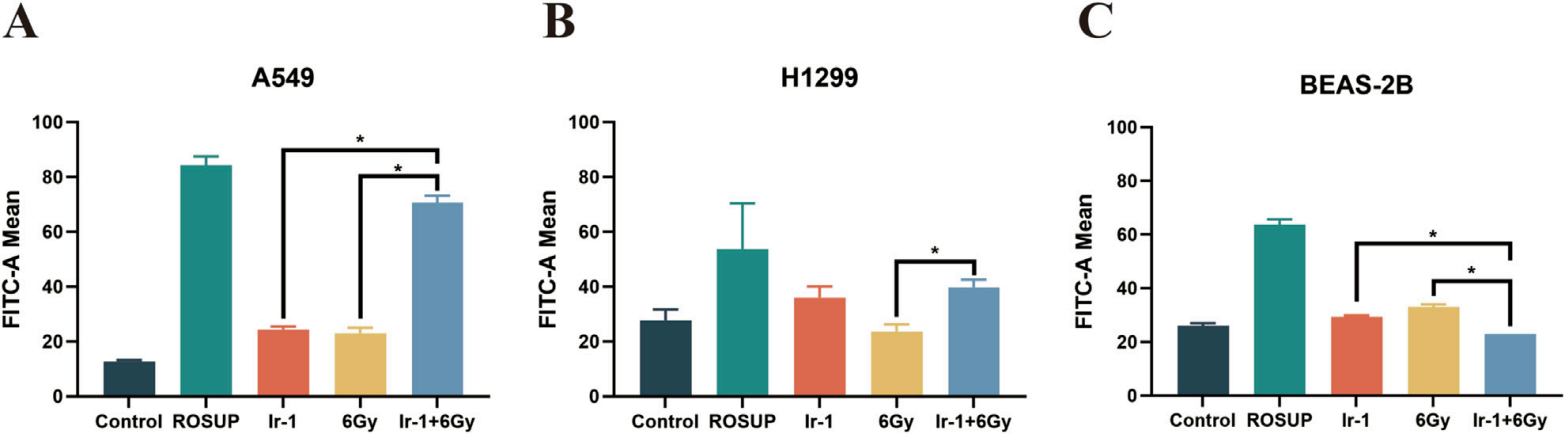
Ir-1 enhanced radiation-induced the ROS generation in lung cancer cells. The A549 **(A)**, H1299 **(B)** and BEAS-2B **(C)** cells were exposed to ROSUP (positive control), 6 µM of Ir-1 in the absence or presence of 6Gy irradiation. Data shown as mean ± SD, n = 3. *P < 0.05.

Mitochondrial membrane potential and ROS production have a strong positive correlation. Therefore, we next evaluated ROS generation in each treatment group. As shown in Figures 7A, B, ROS were significantly increased in the Ir-1 + 6 Gy group compared with the 6 Gy group or Ir-1 group alone in both A549 and H1299 cells. In contrast, no significant change in ROS was observed in BEAS-2B cells (Figure 7C). These findings suggest that the synergistic effect of Ir-1 and radiation in lung cancer cells may be mediated, at least in part, through the ROS pathway.

### 3.8 Ir-1 enhanced radiation-induced apoptotic protein expression

**FIGURE 8:**
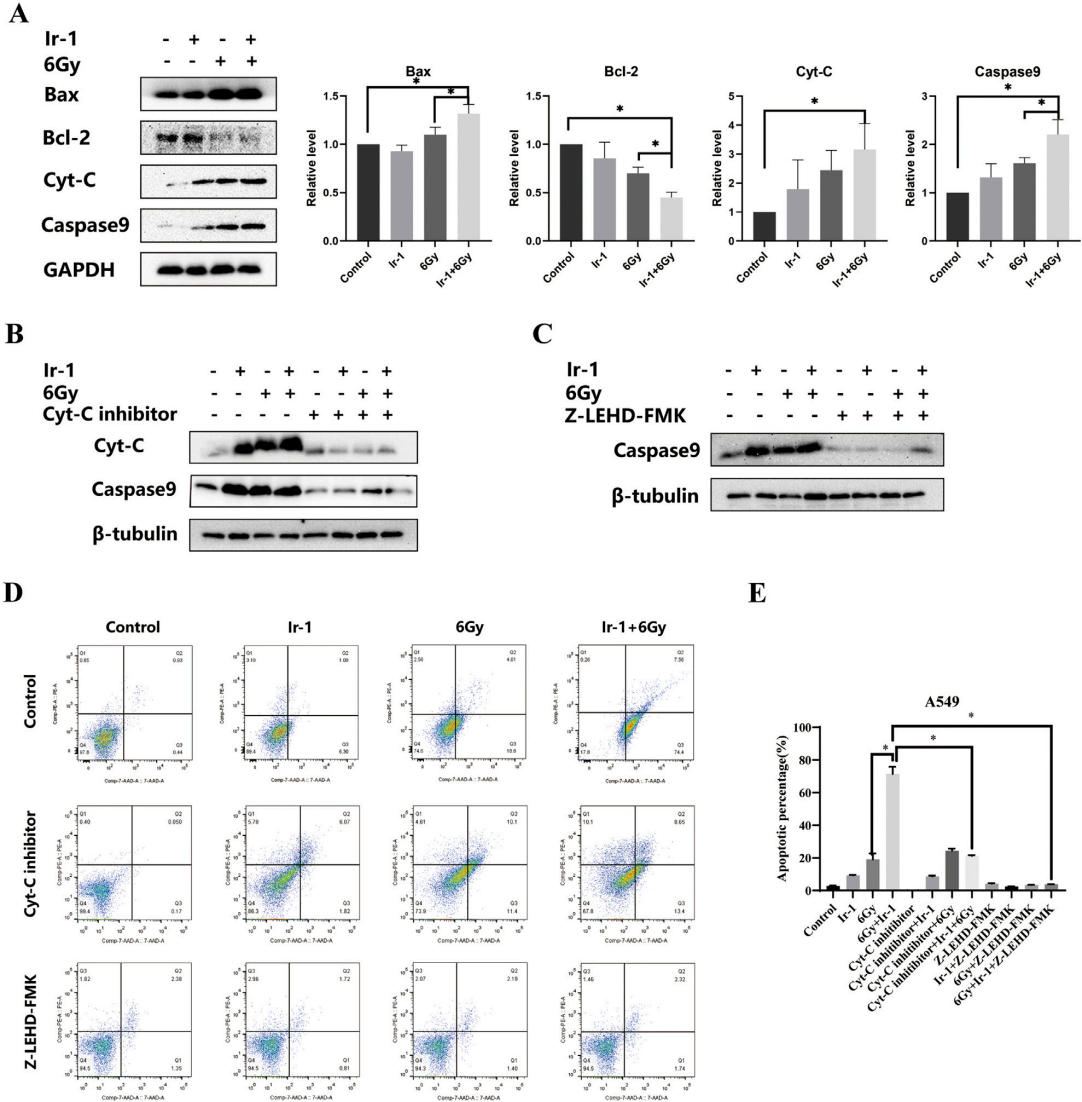
Ir-1 enhanced radiation-induced apoptotic protein expression. **(A)**, Ir-1 and radiation impacted on Bax, Bcl-2, Cyt-C and cleaved-Caspase9 expression. **(B, C)**, the Cyt-C and cleaved-Caspase9 expression level after treated with Cyt-C inhibitor and Caspase9 inhibitor. **(D)**, Representative flow cytometry results of A549 cell apoptosis after indicated treatment. **(E)**, Data analysis from **(D)**. Data shown as mean ± SD, n = 3. *P < 0.05.

To further elucidate the mechanism by which Ir-1 enhances radiation-induced apoptosis, the expression levels of apoptosis-related proteins were analyzed in A549 cells. As shown in Figure 8A, compared with other groups, the expression levels of pro-apoptotic proteins Bax, Cyt-C and cleaved- Caspase9 proteins were significantly increased in Ir-1 + 6 Gy group, while anti-apoptotic protein Bcl-2 level was markedly decreased. To further confirm that Ir-1 enhances the therapeutic effect of radiation through the Cyt-C/Caspase9 signaling pathway, A549 cells were pre-treated with Cyt-C or Caspase9 inhibitors, followed by apoptosis assays. As shown in Figure 8B, Cyt-C inhibitor pre-treatment reduced the expression levels of Cyt-C and cleaved-Caspase9 in the Ir-1 + 6 Gy group. Similarly, Caspase9 inhibitor pre-treatment specifically decreased cleaved-Caspase9 expression levels in the Ir-1 + 6 Gy group (Figure 8C). Flow cytometry was used to detect the apoptosis cells, as shown in Figures 8D, E, the percentage of apoptosis cells was obviously decreased in the inhibitor groups compared with the corresponding groups. These results strongly suggest that Ir-1 enhances radiation-induced apoptosis by activating the mitochondrial apoptosis pathway in lung cancer cells.

## 4 Discussion

Lung cancer remains one of the leading causes of cancer-related incidence and mortality worldwide, with a relatively low 5-year survival rate compared to other major cancers (Huang et al., 2023). CDDP is widely used to be an adjuvant radiotherapy drug. However, platinum drugs have serious cytotoxic effects on normal tissue cells, limit their clinical application (Rosenberg et al., 1969; Santos et al., 2020). Therefore, a critical challenge is to develop novel anticancer agents such as cancer-specific radiosensitizers, that selectively enhance the cytotoxic effects of radiation on tumor cells while minimizing damage to normal tissues (Kuo et al., 2015). In our previous study, we successfully synthesized a novel iridium (III) complex and demonstrated its excellent stability in mice (Li et al., 2019). Building on this foundation, the current study investigated the radiosensitizing potential of this iridium (III) complex in lung cancer cells. Additionally, we investigated the underlying mechanisms by which the iridium (III) complex potentiates the efficacy of radiation therapy against lung cancer cells.

Previous studies had confirmed that some pyridinyl iridium (III) complexes and half sandwich iridium (III) complexes had the antitumor activity (Ma et al., 2019; Zhang et al., 2020; Zhang et al., 2021), primarily through ROS-mediated mitochondrial dysfunction pathway (Wang et al., 2022). The intracellular ROS could induce cell apoptosis, cycle arrest and senescence, while also acting as signaling molecules in the intracellular mitochondria apoptosis pathway. Compared with normal cells, cancer cells exhibit a severely disrupted redox balance, suggesting that ROS regulation could be a potential target for cancer therapy (Moloney and Cotter, 2018). Mitochondria serve as a major source of intracellular ROS and are related to apoptosis induction. They function as key intracellular signaling hubs, emerging as important determinants in cancer development and progression (Guerra et al., 2017). The mitochondrial electron transport chain maintains the stability of mitochondrial membrane potential through redox reaction. The change of mitochondrial membrane potential is an important indicator to evaluate the physiological function of mitochondria (Sakamuru et al., 2022), and an early hallmark of apoptosis. Apoptosis is the natural way of cell physiological death, orderly regulated by apoptosis related signaling pathways, which act as the anticancer therapies trigger apoptosis induction (Mohammad et al., 2015). Radiation-induced DNA damage can trigger G2/M phase arrest in the cell cycle, then affecting cell proliferation (Pawlik and Keyomarsi, 2004). B-cell lymphoma-2 (Bcl-2) family proteins regulate programmed cell death by controlling intracellular signals of apoptosis and participate in the activation of the intracellular mitochondrial apoptosis pathway, including the anti-apoptotic protein Bcl-2 and the pro-apoptotic protein Bax. Bcl-2 and Bax proteins could regulate the release of Cyt-C and then activate the caspase family including Caspase9 and Caspase3, showing caspase cascade reaction and triggering the response of apoptosis process (Guo et al., 2021). Thus, inhibiting cancer cell proliferation and promotion of apoptosis are fundamental strategies for the treatment of cancer. Liao C et al. reported that an iridium (III) complex liposome delivery system increased ROS levels, causing cellular oxidative damage, mitochondrial dysfunction, and inhibiting proliferation (Liao et al., 2018). Huang et al. reported a type of mitochondria-targeted iridium (III) complex possessed enhancing efficacy to restrain the cancer cell proliferation (Nam et al., 2016; Liu et al., 2017). Liu et al. synthesized a kind of polypyridine iridium (III) complex that induces apoptosis through targeting the lysosome and mitochondria (Du et al., 2019). Zhang et al. synthesized three iridium (III) complexes with a new ligand TFBIP entrapped in liposomes, which targeted mitochondria, enhancing intracellular ROS levels and inducing damage to inner and outer membrane structures of mitochondria and release of Cyt-C (Zhang et al., 2022). Chen et al. synthesized two iridium (III) complexes having ligand THPIP, and confirmed that they induced an accumulation of toxic epoxidized lipid MDA and the ROS-mediated mitochondrial dysfunction via activating the PI3K/AKT/mTOR pathway (Chen et al., 2023). Additionally, a luminescent cyclic metallized iridium (III) diimide complex exhibited selective anticancer activity against various cancer cell lines (Lu et al., 2015). Zhou Yi et al. reported that Ir (III)-BBIP promoted apoptosis of A549 cells by regulating the apoptosis signaling pathway (Zhou et al., 2021). Ma et al. comprehensively reviewed a variety of iridium (III) complexes that can target apoptosis of cancer cells (Ma et al., 2019). Compared with previous reports, our study demonstrated a similar trend regarding the antitumor activity of iridium (III) complexes. Although the above studies showed the cytotoxicity of iridium (III) complexes on cancer cells, its effect on apoptosis of normal cells has been less involved. Our results address this gap by providing additional data on the effects of iridium (III) complexes on both lung cancer cells and normal lung epithelial cells, thereby offering a more comprehensive evaluation of their therapeutic potential and selectivity.

In conclusion, the novel iridium (III) complex Ir-1 [Ir (MDQ)2 (acac)] demonstrated to exert potent anticancer effects and enhance radiosensitivity in lung cancer cells. Our findings revealed that Ir-1 significantly augmented the inhibitory effects of radiation by inducing apoptosis, causing G2/M phase cell cycle arrest, inhibiting cell migration and invasion, promoting intracellular ROS production and activating mitochondria apoptosis pathway in lung cancer cells. The findings provided compelling evidence that Ir-1 represents a promising candidate as a selective and effective radiosensitizer for lung cancer treatment, warranting further investigations.

## Data Availability

The raw data supporting the conclusions of this article will be made available by the authors, without undue reservation.
