# Supplementary material for: Radiosensitization effect of iridium (III) complex on lung cancer cells via mitochondria apoptosis pathway
Source: Front Pharmacol. 2025 Mar 27;16:1562228. doi: 10.3389/fphar.2025.1562228 (PMC11994930; doi:10.3389/fphar.2025.1562228)
Supplement: Supplementary file 1 [file Table1.docx]

**Radiosensitization Effect of Iridium (III) Complex on Lung Cancer Cells via Mitochondria Apoptosis Pathway**

**Tables**

**Table S1.** IC_50_ (μM) values of the iridium (III) complexes and CDDP.

| IC_50_ (μM) | A549 | H1299 | HepG2 | Hela | HCT116 | BEAS-2B | MSC |
| --- | --- | --- | --- | --- | --- | --- | --- |
| Ir-1 | 11.16±0.51 | 53.89±5.63 | 16.32±0.21 | 16.47±2.75 | 18.91±0.27 | >200 | >200 |
| Ir-2 | 14.46±2.84 | >200 | 21.54±0.21 | 9.55±0.72 | 179.47±0.96 | >200 | 23.15±0.59 |
| Ir-3 | 15.31±0.57 | 25.61±0.23 | 24.28±0.35 | 36.44±0.35 | 179.67±0.39 | 23.93±0.92 | 3.82±0.12 |
| CDDP | 12.94±0.22 | 6.52±0.39 | 12.93±0.78 | 5.38±0.35 | 9.25±0.55 | 28.13±0.58 | 20.79±0.93 |

IC_50_ value was the drug concentration against selected different cells for 24h necessary for 50% inhibition of cells growth.

**Table S2.** Related parameters of A549 cell Survival Fraction.

| Group | D_0_ | Dq | N | SER |
| --- | --- | --- | --- | --- |
| Control | 2.338 | 1.145 | 1.632 |  |
| Ir-1 | 1.468 | 0.729 | 1.643 | 1.592 |
| CDDP | 2.127 | 1.021 | 1.616 | 1.099 |

**Table S3.** Related parameters of H1299 cell Survival Fraction.

| Group | D_0_ | Dq | N | SER |
| --- | --- | --- | --- | --- |
| Control | 1.719 | 2.215 | 3.628 |  |
| Ir-1 | 1.443 | 0.946 | 1.926 | 1.191 |
| CDDP | 1.510 | 1.730 | 3.140 | 1.138 |

**Table S4.** Related parameters of BEAS-2B cell Survival Fraction.

| Group | D_0_ | Dq | N | SER |
| --- | --- | --- | --- | --- |
| Control | 0.8518 | 2.221 | 13.56 |  |
| Ir-1 | 0.8489 | 2.007 | 10.63 | 1.003 |
| CDDP | 0.7164 | 1.882 | 13.84 | 1.192 |

**Table S5.** Related parameters of MSC cell Survival Fraction.

| Group | D_0_ | Dq | N | SER |
| --- | --- | --- | --- | --- |
| Control | 1.785 | 1.126 | 4.878 |  |
| Ir-1 | 1.778 | 1.320 | 3.846 | 1.003 |

**Table S6.** Cell cycle with different treatments of A549 cells.

| Group | G0/G1 | S | G2/M |
| --- | --- | --- | --- |
| Control | 63.23±1.09 | 27.39±0.90 | 9.38±0.43 |
| Ir-1 (6μM) | 72.32±0.17 | 15.52±1.82 | 12.16±1.67 |
| 6Gy | 78.24±1.18* | 5.17±0.98* | 16.59±0.28* |
| Ir-1+6Gy | 71.48±1.25* | 4.37±0.24* | 23.79±1.36* |
| CDDP | 60.84±1.78 | 17.54±0.41 | 21.95±1.59 |
| CDDP+6Gy | 62.68±3.34 | 15.415±0.40 | 21.90±3.74 |

**P<0.05* vs. counterpart treatment.

**Table S7.** Cell cycle with different treatments of H1299 cells.

| Group | G0/G1 | S | G2/M |
| --- | --- | --- | --- |
| Control | 63.06±1.10 | 30.77±3.09 | 6.17±2.04 |
| Ir-1 (10μM) | 56.47±2.91 | 31.41±1.50 | 13.64±0.23 |
| 6Gy | 52.69±1.24* | 30.70±1.69 | 16.60±1.05* |
| Ir-1+6Gy | 47.97±1.03* | 31.29±1.50 | 20.72±0.56* |
| CDDP | 52.60±1.64 | 30.46±2.35 | 16.90±0.82 |
| CDDP+6Gy | 44.09±2.96 | 32.27±0.82 | 23.65±3.22 |

**P<0.05* vs. counterpart treatment.

**Table S8.** Cell cycle with different treatments of BEAS-2B cells.

| Group | G0/G1 | S | G2/M |
| --- | --- | --- | --- |
| Control | 75.42±1.79 | 13.94±1.67 | 10.63±0.68 |
| Ir-1 (6μM) | 75.47±0.35 | 12.31±0.78 | 12.00±1.08 |
| 6Gy | 58.24±1.18* | 25.17±0.98* | 16.59±0.28 |
| Ir-1+6Gy | 50.36±2.89* | 32.35±0.99* | 17.29±1.97 |

**P<0.05* vs. counterpart treatment.

**Images uploaded to jianguoyun**

https://www.jianguoyun.com/p/DTvpisEQo9uZDRjq6-gFIAA
